# The risk factors of gestational diabetes mellitus in patients with polycystic ovary syndrome: What should we care

**DOI:** 10.1097/MD.0000000000026521

**Published:** 2021-08-06

**Authors:** Xiaocui Li, Xinru Liu, Yan Zuo, Jiejun Gao, Yan Liu, Wei Zheng

**Affiliations:** a Department of Obstetrics and Gynecology, West China Second Hospital of Sichuan University, Chengdu, China; b Key Laboratory of Birth Defects and Related Diseases of Women and Children (Sichuan University), Ministry of Education Chengdu, China.

**Keywords:** care, gestational diabetes mellitus, management, PCOS, pregnancy

## Abstract

The influencing factors of gestational diabetes mellitus (GDM) in the polycystic ovary syndrome (PCOS) patients remain unclear, we aimed to investigate the risk factors of GDM in patients with PCOS, to provide reliable evidence for the prevention and treatment of GDM in PCOS patients.

PCOS patients treated in our hospital from January 1, 2019 to October 31, 2020 were included. The personal and clinical treatment details of GDM and no GDM patients were analyzed. Logistic regressions were performed to analyze the factors influencing the occurrence of GDM.

A total of 196 PCOS patients were included, the incidence of GDM in patients with PCOS was 23.98%. There were significant differences in the age, body mass index, insulin resistance index, fasting insulin, testosterone, androstenedione, and sex hormone-binding protein between GDM and no GDM patients with PCOS (all *P* < .05), and no significant differences in the family history of GDM, the history of adverse pregnancy, and multiple pregnancies were found (all *P* > .05). Age ≥30 years (odds ratio (OR) 2.418, 95% confidence interval (CI) 1.181–3.784), body mass index ≥24 kg/m^2^ (OR 1.973, 95%CI 1.266–3.121), insulin resistance index ≥22.69 (OR 2.491, 95%CI 1.193–4.043), fasting insulin ≥22.71 mIU/L (OR 2.508, 95%CI 1.166–5.057), testosterone ≥2.85 nmol/L (OR 1.821, 95%CI 1.104–2.762), androstenedione ≥6.63 nmol/L (OR 1.954, 95%CI 1.262–2.844), sex hormone-binding protein <64.22 nmol/L (OR 1.497, 95%CI 1.028–2.016) were the independent risk factors of GDM in patients with PCOS (all *P* < .05). The incidence of preeclampsia, premature delivery, premature rupture of membranes, polyhydramnios, and postpartum hemorrhage in the GDM group was significantly higher than that of the no-GDM group (all *P* < .05). There was no significant difference in the incidence of oligohydramnios between the 2 groups (*P* = .057).

The incidence of GDM in PCOS patients is high, and the measures targeted at the risk factors are needed to reduce the occurrence of GDM in patients with PCOS.

## 1 Introduction

Polycystic ovary syndrome (PCOS) is a commonly seen disease in clinical gynecology.^[1]^ Most patients with PCOS present with infertility due to ovulation dysfunction.^[2]^ After treatment with menstrual cycle adjustment, ovulation induction, and assisted reproductive technology, most patients can become pregnant, but due to the influence of underlying diseases, although the pregnancy can be successful, the adverse pregnancy outcome and the incidence of complications during pregnancy is relatively high.^[3,4]^ Studies^[5,6]^ have shown that the incidence of diabetes in PCOS women between 20 and 40 years old is as high as 20% to 40%. Although PCOS's role in increasing the risk of diabetes has long been proposed, the relationship between PCOS and diabetes lacks a large sample of data. Studies^[7,8]^ have shown that metabolic screening is necessary for PCOS women in early pregnancy. Early screening and identification can not only strengthen maternal-fetal monitoring but also reduce the incidence of maternal and infant complications affected by PCOS.^[9]^

PCOS is a common female endocrine and metabolic disorder, which has been found that PCOS patients are prone to gestational diabetes mellitus (GDM).^[10]^ The core mechanism may be related to insulin resistance (IR).^[11]^ The secretion of pregnancy hormones such as estrogen, progesterone, and prolactin in PCOS patients increases after pregnancy, which leads to a further increase in the degree of IR and aggravates the risk of glucose metabolism disorders in patients.^[12]^ Many previous studies^[13–15]^ have pointed out that patients with PCOS have a significantly increased risk of gestational diabetes, preeclampsia, and hypertension during pregnancy, and the risk of preterm birth, neonatal hypoglycemia, and neonatal hyperbilirubinemia also significantly increased. The early identification of GDM women in patients with PCOS is vital to the prevention of short- and long-term consequences of GDM, especially to prevent fetal programming and long-term health consequences for offspring.^[16]^ Therefore, we aimed to identify the potential risk factors of GDM in patients with PCOS, to provide reliable evidence to the prevention of GDM and management of PCOS.

## 2 Methods

### 2.1 Ethical consideration

Our study was conducted in a tertiary hospital (Chengdu, Sichuan province) of China. The procedure followed in this study was in compliance with the ethical standards set by the relevant ethics committee of our hospital, and it was approved by the ethics committee of West China Second Hospital of Sichuan University (approval number: TSR190094-2A). All pregnant women included in this study were well informed of the research purpose and details, and all the included patients signed the written informed consent.

### 2.2 Patients

This study selected PCOS patients who were treated in our hospital from January 1, 2019 to October 31, 2020 as the research subjects. The inclusion criteria were as follows: the woman was pregnant and with age >18 years; the patients had been checked and diagnosed as PCOS accordingly; the patients underwent follow-up prenatal monitoring and delivery in our hospital; the patients had been well informed and agreed to participants in this study. The patients were excluded if they refused to participate.

### 2.3 Diagnosis definition

The diagnostic criteria of PCOS referred to the relevant international PCOS diagnostic criteria:^[17,18]^ (1) sparse ovulation or anovulation; (2) clinical manifestations of hyperandrogen and/or hyperandrogenemia; (3) polycystic ovarian manifestations: ultrasound indicates the diameter of 1 or both sides of the ovary 2 to 9 mm follicles ≥12, and/or ovarian volume ≥10 mL; and (4) if 2 of the 3 items are met and other high androgen causes, such as congenital adrenal hyperplasia, Cushing syndrome are excluded, then the patient can be diagnosed as having PCOS.

The diagnostic criteria of GDM referred to the relevant diagnostic criteria in China:^[19]^ pregnant women would undergo a 75 g oral glucose tolerance test (OGTT) fasting blood glucose test at 24 to 28 weeks of gestation, and the blood glucose threshold values of 0, 1, and 2 hours after taking the sugar were 5.1, 10.0, 8. 5 mmol/L. GDM was diagnosed if the blood glucose level met or exceeded the above standards at any point; if the OGTT was normal for the first time, we would repeat OGTT in the third trimester if necessary.

### 2.4 Data collections

Two authors independently collected the personal and clinical treatment details of included patients, including age, body mass index (BMI), family history of hypertension, family history of GDM, the history of adverse pregnancy, multiple pregnancies, insulin resistance index (HOMA-IR), fasting insulin (Fins), testosterone, androstenedione, sex hormone-binding protein (SHBG). The delivery details were also collected, including preeclampsia, premature delivery, premature rupture of membranes, polyhydramnios, oligohydramnios, and postpartum hemorrhage.

### 2.5 Statistical analysis

We used SPSS 22.0 statistical software to perform statistical analysis on the data. Measurement data were expressed as mean ± standard deviation (*x* ± *s*), and *t* test was used for comparison between groups. Binary data were displayed as percentages (%), and the Chi-square test was used for comparison between groups. Logistic regressions were used to analyze the related factors influencing the occurrence of GDM. All hypothesis testing used two-sided testing, and *P* < .05 meant that the difference between groups was statistically significant.

## 3 Results

### 3.1 The characteristics of included patients

A total of 196 PCOS patients were included, of whom 47 patients had suffered from GDM, the incidence of GDM in patients with PCOS was 23.98%. As presented in Table 1, there were significant differences in the age, BMI, HOMA-IR, Fins, testosterone, androstenedione, and SHBG between GDM and no GDM patients with PCOS (all *P* < .05). The age, BMI, HORMA-IR, level of Fins, testosterone, androstenedione in the GDM patients were significantly higher than that of no GDM patients, and the level of SHBG in the GDM patients was significantly lower than that of no GDM patients. No significant differences in the family history of GDM, the history of adverse pregnancy, and multiple pregnancies between GDM and no GDM patients with PCOS were found (all *P* > .05).

**Table 1:** The characteristics of included PCOS patients,.

Variables	GDM group (n = 47)	No-GDM group (n = 149)	*t*/χ^2^	*P*
Age (yrs)	32.81 ± 8.15	27. 05 ± 8.46	1.231	.007
BMI (kg/m^2^)	25.93 ± 2.44	22.63 ± 1.94	1.129	.014
Family history of hypertension	12 (25.53%)	36 (24.16%)	1.273	.082
Family history of GDM	11 (23.40%)	29 (19.46%)	1.622	.059
The history of adverse pregnancy	9 (19.15%)	22 (14.77%)	1.417	.066
Multiple pregnancy	11 (23.40%)	26 (17.45%)	1.899	.051
HOMA-IR	3.22 ± 1.25	1.08 ± 0.74	1.024	.019
Fins (mIU/L)	24.19 ± 2.17	21.98 ± 1.94	6.187	.033
Testosterone (nmol/L)	3.27 ± 1.36	1.95 ± 1.02	1.224	.047
Androstenedione (nmol/L)	8.63 ± 2.77	4.13 ± 1.29	2.213	.036
SHBG (nmol/L)	57.44 ± 9.23	67.17 ± 10.23	3.104	.009

### 3.2 The risk factors of HDCP in patients with PCOS

The variable assignments of multivariate logistic regression were presented in Table 2. As Table 3 indicated, logistic regression analyses had found that age ≥30 years (odds ratio (OR) 2.418, 95% confidence interval (CI) 1.181–3.784), BMI ≥24 kg/m^2^ (OR 1.973, 95%CI 1.266–3.121), HOMA-IR ≥22.69 (OR 2.491, 95%CI 1.193–4.043), Fins ≥22.71 mIU/L (OR 2.508, 95%CI 1.166–5.057), testosterone ≥2.85 nmol/L (OR 1.821, 95%CI 1.104–2.762), androstenedione ≥6.63 nmol/L (OR 1.954, 95%CI 1.262–2.844), and SHBG <64.22 nmol/L (OR 1.497, 95%CI 1.028–2.016) were the independent risk factors of GDM in patients with PCOS (all *P* < .05).

**Table 2:** The variable assignment of multivariate logistic regression.

Factors	Variables	Assignment
GDM	*Y*	Yes = 1, no = 2
Age (yrs)	*X*_1_	≥30 = 1, <30 = 2
BMI (kg/m^2^)	*X*_2_	≥24 = 1, <24 = 2
HOMA-IR	*X*_3_	≥22.69 = 1, <2.69 = 2
Fins (mIU/L)	*X*_4_	≥22.71 = 1, <22.71 = 2
Testosterone (nmol/L)	*X*_5_	≥2.85 = 1, <2.85 = 2
Androstenedione (nmol/L)	*X*_6_	≥6.63 = 1, <6.63 = 2
SHBG (nmol/L)	*X*_7_	<64.22 = 1, ≥64.22 = 2

**Table 3:** Logistic regression analysis on the risk factors of GDM in patients with PCOS.

	Unadjusted			Adjusted		
Variables	OR	95%CI	*P*	OR	95%CI	*P*
Age ≥30 yrs	5.124	3.081–7.224	.042	2.418	1.181–3.784	.032
BMI ≥24 kg/m^2^	3.118	2.143–5.125	.012	1.973	1.266–3.121	.021
HOMA-IR ≥22.69	4.233	1.768–6.007	.024	2.491	1.193–4.043	.015
Fins ≥22.71 mIU/L	3.741	2.125–6.225	.016	2.508	1.166–5.057	.022
Testosterone ≥2.85 nmol/L	2.217	1.206–4.166	.039	1.821	1.104–2.762	.013
Androstenedione ≥6.63 nmol/L	3.102	2.147–5.124	.017	1.954	1.262–2.844	.046
SHBG <64.22 nmol/L	2.197	1.284–3.179	.025	1.497	1.028–2.016	.039

### 3.3 The pregnancy outcomes

As presented in Table 4, the incidence of preeclampsia, premature delivery, premature rupture of membranes, polyhydramnios, and postpartum hemorrhage in the GDM group was significantly higher than that of the no-GDM group (all *P* < .05), there was no significant difference in the incidence of oligohydramnios between the 2 groups (*P* = .057).

**Table 4:** Comparison of pregnancy outcomes between GDM and no-GDM PCOS patients.

Variables	GDM group (n = 47)	No-GDM group (n = 149)	*t*/χ^2^	*P*
Preeclampsia	9 (19.15%)	4 (2.69%)	1.502	.002
Premature delivery	7 (14.89%)	5 (3.35%)	1.117	.024
Premature rupture of membranes	8 (17.02%)	4 (2.69%)	1.381	.012
Polyhydramnios	5 (10.64%)	4 (2.69%)	1.212	.039
Oligohydramnios	2 (4.26%)	5 (3.35%)	1.166	.057
Postpartum hemorrhage	4 (8.51%)	2 (1.34%)	1.201	.003

## 4 Discussions

The prevalence of GDM is higher than 30% in high-risk pregnant women and PCOS women are part of this high-risk pregnant women population.^[20,21]^ The incidence of GDM in PCOS patients has reached about 32.17%.^[22]^ With the changes in human life patterns and survival styles, the incidence of GDM complicated by PCOS is increasing, and the early prediction, prevention, and treatment of GDM in PCOS patients are urgent.^[23]^ The onset of GDM involves factors such as heredity, immunity, hormones, trace elements, mood, hypertension, etc, but there are no reliable predictors.^[24]^ The results of our study have found that the incidence of GDM in patients with PCOS was 23.98%, and age ≥30 years, BMI ≥24 kg/m^2^, HOMA-IR ≥22.69, Fins ≥22.71 mIU/L, testosterone ≥2.85 nmol/L, androstenedione ≥6.63 nmol/L, and SHBG <64.22 nmol/L were the independent risk factors of GDM in patients with PCOS, and GDM is closely associated with the adverse delivery outcomes. Clinically, early prevention and intervention measures should be taken for these risk factors to improve the prognosis of PCOS patients.

After the occurrence of PCOS, hyperinsulinemia interacts with insulin-like growth factors in the ovary, causing the follicular membrane cells to fail to convert androstenedione into estrogen, which increases androgens and forms hyperandrogenemia.^[25,26]^ Studies^[27–29]^ have found that androgens can promote the apoptosis of pancreatic β cells. SHBG is a glycoprotein produced by the liver that specifically binds and transports sex hormones and regulates the concentration of sex hormones in the blood.^[30]^ In humans, SHBG mainly binds testosterone, followed by estradiol. Insulin is an important regulator of SHBG metabolism.^[31]^ In the presence of IR, insulin sensitivity decreases, and insulin secretion increases compensatively. Increased insulin levels can inhibit the production of SHBG in the liver and reduce the synthesis of SHBG in the liver.^[32,33]^ The decrease of SHBG in the circulatory system makes the level of sex hormones disorder, causing glucose and fat metabolism disorders, further aggravating insulin resistance, and leading to the occurrence of GDM.^[34,35]^

Age factor is an important factor affecting the onset of gestational diabetes. Studies^[36,37]^ have found that with the increase of age, the incidence of GDM gradually increases in pregnant women aged 25–40. Studies^[38,39]^ have reported that compared with pregnant women aged 20 to 30, PCOS patients over 30 years of age are at increased risk of gestational diabetes. Studies^[40,41]^ have shown that body mass index is closely related to the occurrence of gestational diabetes in PCOS patients. Previous studies^[42,43]^ have shown that increased BMI is a risk factor for GDM, which is consistent with the results of this study. It has been reported that 30% to 70% of PCOS patients have IR, the sensitivity of peripheral tissues to insulin decreases, the biological effect of insulin is lower than normal, and the body produces compensatory hyperinsulinemia to counteract IR.^[44]^ In addition, during pregnancy, changes in the body's hormones lead to decreased insulin sensitivity, and placental insulinase secreted by the placenta accelerates the degradation of insulin in the body, which can also cause IR.^[45,46]^ Therefore, when the amount of insulin secretion is not enough to meet the needs of IR, that is, when insulin secretion is decompensated, fasting and abnormal glucose tolerance occur during pregnancy in PCOS patients, leading to the occurrence of gestational diabetes.^[47,48]^ The results of this study show that the detection of IR and Fins in PCOS patients has important guiding significance for predicting the risk of PCOS complicated by GDM.

Previous studies^[49,50]^ have found that the FINS and HOMA-IR levels of pregnant women in the PCOS group were significantly higher than those in the non-PCOS group and the control group. Compared with the GDM alone group, the PCOS combined GDM group used more insulin.^[51]^ Because the BMI of pregnant women in the PCOS group is relatively high, excess fat in the body changes insulin secretion and sensitivity, and reduces the number of insulin receptors in fat, liver, and muscle tissue cells, which in turn affects the body's downregulation and leads to insulin secretion, eventually resulting in an increase in FINS and IR.^[52,53]^ Additionally, a previous study^[54]^ showed that the incidence of preeclampsia, premature delivery, and polyhydramnios in the GDM group was significantly higher than that of the no-GDM group and the control group, which is consistent with our findings. Previous studies^[13,55]^ have found that there is a strong correlation between PCOS and preterm birth. Therefore, GDM is a high-risk factor that increases adverse pregnancy outcomes in pregnant women with PCOS. Early diagnosis, active prevention, and treatment should be taken to reduce adverse pregnancy outcomes.

The limitations of the study should be concerned. Firstly, the sample size is small, it may underpower to detect the potential influencing factors, future studies with a larger sample size are needed. Secondly, our study is a retrospective design, the collected data are limited, many other variables that may influence the GDM development should be further elucidated. Thirdly, the women with GDM without PCOS cannot be included for group comparison in this study, the controlled variables between groups would make the results more reliable and convincing, which warrants further investigation in future studies.

## 5 Conclusions

In summary, PCOS patients are a high-risk group of GDM during pregnancy. Older age, larger BMI, higher HOMA-IR, Fins, testosterone, androstenedione, and low SHBG are the independent risk factors for GDM in PCOS patients. Therefore, age, BMI, HOMA-IR, Fins, testosterone, androstenedione, and SHBG are important predictors of gestational diabetes in PCOS patients before pregnancy. At the same time, the adverse effects of GDM on maternal and infant outcomes should be given high priority. In clinical work, we should strengthen the popularization and education of pre-pregnancy-related knowledge of PCOS patients and the management and control measures during post-pregnancy check-ups to achieve early prevention, timely diagnosis of GDM.

## Author contributions

XL designed research; XL, YZ, JG, YL, and WZ conducted research; XL and YZ analyzed data; XL wrote the first draft of the manuscript; and XL had primary responsibility for final content. All authors read and approved the final manuscript.

**Conceptualization:** Xiaocui Li, Yan Zuo, Jiejun Gao, Wei Zheng.

**Data curation:** Xiaocui Li, Jiejun Gao, Yan Liu.

**Formal analysis:** Yan Zuo, Wei Zheng.

**Investigation:** Xiaocui Li, Xinru Liu, Yan Zuo, Jiejun Gao, Yan Liu, Wei Zheng.

**Methodology:** Jiejun Gao, Yan Liu.

**Project administration:** Xinru Liu, Yan Zuo, Jiejun Gao.

**Resources:** Xinru Liu, Yan Zuo, Jiejun Gao, Yan Liu, Wei Zheng.

**Software:** Xinru Liu, Yan Zuo, Yan Liu.

**Supervision:** Xiaocui Li.

**Validation:** Xiaocui Li, Xinru Liu.

**Visualization:** Xiaocui Li.

**Writing – original draft:** Xiaocui Li.
